# Conversion of NaHCO_3_ to Na_2_ CO_3_ with a growth of *Arthrospira platensis* cells in 660 m^2^ raceway ponds with a CO_2_ bicarbonation absorber

**DOI:** 10.1111/1751-7915.13497

**Published:** 2019-10-23

**Authors:** Wangbiao Guo, Jun Cheng, Kubar Ameer Ali, Santosh Kumar, Caifeng Guo

**Affiliations:** ^1^ State Key Laboratory of Clean Energy Utilization Zhejiang University Hangzhou 310027 China; ^2^ Ordos Jiali Spirulina Co., Ltd Ordos 016199 China

## Abstract

The weight ratio of Na_2_ CO_3_/NaHCO_3_ was investigated in order to improve microalgal productivity in large‐scale industrial operations by converting NaHCO_3_ to Na_2_ CO_3_ with a growth of *Arthrospira platensis* cells in 660 m^2^ raceway ponds. Two microalgal cultivation systems with a NaHCO_3_ by‐product (SPBP) and a CO_2_ bicarbonation absorber (CBAP) were firstly thoroughly introduced. There was a 13.3% decrease in the initial weight ratio of Na_2_ CO_3_/NaHCO_3_ resulting in a 25.3% increase in the biomass growth rate with CBAP, compared to that of SPBP. Increased sunlight intensity, solution temperature and pH all resulted in both a higher ${\text{HCO}}_{3}^{-}$ absorbance and ${\text{CO}}_{3}^{2-}$ release, thereby increasing the weight ratio of Na_2_ CO_3_/NaHCO_3_ during the growth of *A. platensis*. The biomass growth rate was peaked at 39.9 g m^−2^ day^−1^ when the weight ratio of Na_2_ CO_3_/NaHCO_3_ was 3.7. Correspondingly, the cell pigments (chlorophyll *a* and carotenoid) and trichome size (helix pitch and trichome length) reached to a maximum state of 8.47 mg l^−1^, 762 μg l^−1^, 57 and 613 μm under the CBAP system.

## Graphical abstract

The weight ratio of Na_2_CO_3_/NaHCO_3_ was investigated in order to improve microalgal productivity in large‐scale industrial operations. Two microalgal cultivation systems with a NaHCO3 by‐product (SPBP) and a CO_2_ bicarbonation absorber (CBAP) were firstly thoroughly introduced. There was a 13.3% decrease in the initial weight ratio of Na_2_CO_3_/NaHCO_3_ resulting in a 25.3% increase of the biomass growth rate with CBAP, compared to that of SPBP.

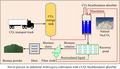

## Introduction

Flue gas emissions of CO_2_ are becoming a serious global problem. In 2017, the concentration of greenhouse gases in the atmosphere reached 407 ppm. Meanwhile, the average annual global temperature has increased by 1.1°C from pre‐industrial times. Therefore, reducing CO_2_ flue gas emissions to control global warming is a crucial task (Zhou *et al*., 2018). Microalgae contributes to approximately one‐third of the global CO_2_ fixation because of their substantial growth rate (Mackinder *et al*., 2017; Rosenzweig *et al*., 2017). Using CO_2_ from flue gases as a carbon source to cultivate microalgae is one alternative method for reducing flue gas emissions.

The CO_2_‐concentrating mechanism (CCM) in the pyrenoid organelle of microalgal cells is the primary reason for the high growth rate of microalgae (Amc *et al*., 2017; Hwangbo *et al*., 2018). The CCM enables microalgal cells to utilize the bicarbonate ions (${\text{HCO}}_{3}^{-}$ ) as a carbon source to avoid CO_2_ limitations in the natural atmosphere (Badger and Price, 2010). Previous studies have suggested that ${\text{HCO}}_{3}^{-}$ can be used by microalgal cells in two ways. In the first process, ${\text{HCO}}_{3}^{-}$ sequentially passes through the plasma membrane, the chloroplast envelope and then the thylakoid membrane, finally arriving at the thylakoid lumen where it is catalysed to CO_2_. CO_2_ is next fixed by the RubisCO enzyme (Amc *et al*., 2017) and then channelled into the Calvin cycle (Spalding, 2008; Mackinder *et al*., 2017; Hwangbo *et al*., 2018). In the second process, ${\text{HCO}}_{3}^{-}$ in the periplasmic space is converted by carbonic anhydrase into CO_2_, which then diffuses to the stroma and is captured by RubisCO. Therefore, enhancing the ${\text{HCO}}_{3}^{-}$ concentration in the microalgal solution is a theoretically feasible method to accelerate the microalgal growth and CO_2_ fixation rates.

To date, approximately 250 hectares of raceway ponds have been established to cultivate *Arthrospira platensis* in Inner Mongolia, China. This is thought to be the largest industrial *A. platensis* cultivation area in China. Approximately 3000 tons of *A. platensis* powder are produced annually and subsequently sold to Europe, America, Australia and Japan. However, few studies have reported on the activities in this area. An NaHCO_3_ plant in this area produces large amounts of by‐products consisting of 40–70% NaHCO_3_ and 5–10% Na_2_CO_3_, which are suitable raw materials for the microalgal cultivation industry (Lu *et al*., 2011; Toyoshima *et al*., 2015; Chen *et al*., 2016; Yuan *et al*., 2018). However, after several years of development, numerous problems have become apparent when using NaHCO_3_ by‐products as a carbon source. First, because of the low mixing and mass transfer efficiencies, the ${\text{HCO}}_{3}^{-}$ concentration is too low for higher *A. platensis* growth. Second, the operational cost, especially labour, is high. For example, there are approximately 3800 raceway ponds in the whole area. Workers need to add NaHCO_3_ by‐products to each raceway pond daily, which is a huge labour cost. Moreover, the NaHCO_3_ is expensive, and enterprises need to be able to afford the raw materials. Third, the ash content of the microalgal powder is relatively high because the NaHCO_3_ by‐product contains many impurities, such as sand and soil. This reduces the commercial value of the *A. platensis* powder. Fourth, after cultivation, the *A. platensis* solution has a high concentration of Na_2_CO_3_, resulting in a high pH that is harmful to the environment, thereby hindering the development of the *A. platensis* industry. Thus, there is a search for alternative methods of *A. platensis* cultivation.

Based on the above information, a CO_2_ bicarbonation absorber (CBA) was developed to improve ${\text{HCO}}_{3}^{-}$ concentrations in the *A. platensis* solutions. The CBA uses CO_2_ gas and the Na_2_CO_3_ solution to produce NaHCO_3_ to stimulate *A. platensis* growth (CO_2_ + ${\text{CO}}_{3}^{2-}$ + H_2_O → 2${\text{HCO}}_{3}^{-}$ ). This method not only reduces the operational cost but also improves the CO_2_ utilization efficiency. A previous lab‐scale study investigated the reaction time, reaction pressure, initial Na_2_CO_3_ solution and solution volume ratio of the CBA process (CBAP) to optimize the molar proportion of ${\text{HCO}}_{3}^{-}$/${\text{CO}}_{3}^{2-}$ (Guo *et al*., 2019). Those results showed that the microalgal growth rate increased by a factor of 5.0 at an initial molar ${\text{HCO}}_{3}^{-}$/${\text{CO}}_{3}^{2-}$ proportion of 92% compared with normal conditions (atmosphere pressure and room temperature). During industrial applications of CBA, we accidently found that the weight ratio of Na_2_CO_3_/NaHCO_3_ (WRB) in the residual solution is an important parameter for estimating *A. platensis* growth. The cell pigments and trichome size are closely affected by the WRB. However, to our knowledge, no previous research has considered the influence of the WRB on microalgal growth.

## Results and discussion

### Investigating the weight ratio of Na_2_CO_3_/NaHCO_3_

**Figure 1:**
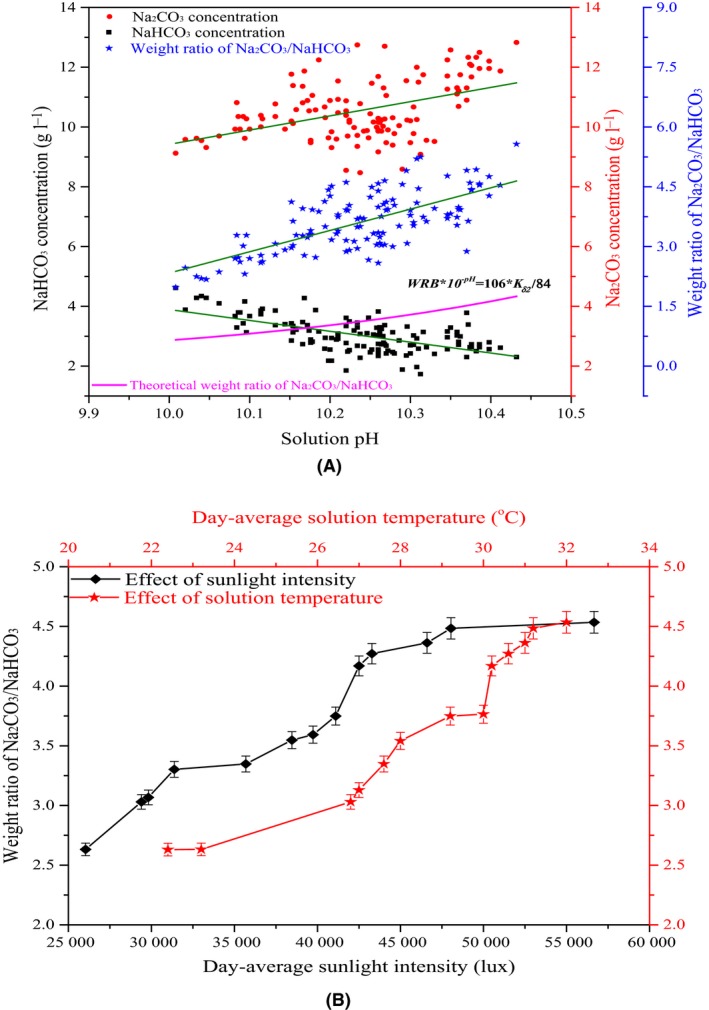
Effects of environmental conditions on Na_2_ CO_3_/NaHCO_3_ weight ratio in residual solution after *Arthrospira platensis* growth in 660 m^2^ open raceway ponds. A. Effects of solution pH. B. Effects of sunlight intensity and solution temperature.

The solution pH, NaHCO_3_ and Na_2_CO_3_ concentrations in the residual solution of the four raceway ponds were recorded (Fig. 1A). The data showed that with increasing solution pH, the Na_2_CO_3_ concentration gradually increased while the NaHCO_3_ concentration decreased. It is evident that OH^−^ is continually excreted during the growth of the *A. platensis* cells. The higher OH^−^ concentration promotes the following reaction: OH^−^ + ${\text{HCO}}_{3}^{-}$ → ${\text{CO}}_{3}^{2-}$ + H_2_O. According to Henry's law (Al‐Anezi *et al*., 2008; Morton, 2008), when the pH is between 10 and 12, the molar ratio of ${\text{CO}}_{3}^{2-}$/TIC (the mixture of ${\text{HCO}}_{3}^{-}$ , ${\text{CO}}_{3}^{2-}$ and H_2_CO_3_) increases continually with increasing pH, while the ratio of ${\text{HCO}}_{3}^{-}$/TIC decreases. Theoretically, the weight ratio of Na_2_CO_3_/NaHCO_3_(WRB) and the solution pH follows the function of *WRB × 10* ^*−pH*^ *= 106 × K*_*σ2*_ */84*(*K*_*σ2*_ *= *10^−10.29^). Meanwhile, ${\text{CO}}_{3}^{2-}$ */TIC = K*_*σ1*_ *K*_*σ2*_ */(K*_*σ1*_ *K*_*σ2*_ *+ K*_*σ1*_ *[H* ^*+*^ *] + [H* ^*+*^ *]* ^*2*^ *)*, ${\text{HCO}}_{3}^{-}$ */TIC = K*_*σ1*_ *[H* ^*+*^ *]/(K*_*σ1*_ *K*_*σ2*_ *+ K*_*σ1*_ *[H* ^*+*^ *] + [H* ^*+*^ *]* ^*2*^ *)*, where *K*_*σ1*_ and *K*_*σ2*_ are equilibrium constants. Therefore, in theory, the WRB increases with the solution pH. However, the theoretical WRB is far less than what was observed in the experimental data. The reasons for this phenomenon are threefold. First, the theoretical WRB only considers the dissolution equilibrium of ${\text{HCO}}_{3}^{-}$ and ${\text{CO}}_{3}^{2-}$. The measured WRB depends on the chemical equilibrium and microalgal growth. Second, ${\text{HCO}}_{3}^{-}$ is the primary carbon source of *A. platensis* cells. With the growth of *A. platensis*, ${\text{HCO}}_{3}^{-}$ is continually consumed, while ${\text{CO}}_{3}^{2-}$ is continually produced. Thus, the *A. platensis* cells caused the higher experimental WRB. Third, the TIC increases with the growth of *A. platensis. *The theoretical WRB assumes that the TIC is constant; however, in practice the TIC increased gradually and was higher than the theoretical TIC. Therefore, the higher solution pH resulted in a gradual increase in the WRB and the WRB is closely affected by the pH of the residual solution.

In addition to the residual solution pH, the daily average sunlight intensity and microalgal solution temperature also affect the WRB. As the sunlight intensity and solution temperature simultaneously increased from 26 040 to 56 660 lux and 22.4 to 32.0°C, respectively, the WRB gradually increased from 2.6 to 4.5 (Fig. 1B). It is believed that the sunlight intensity substantially affects photosynthesis and the solution temperature affects enzymatic activities (Gao and Zengling, 2008; Béchet *et al*., 2017; Cheng *et al*., 2018a). An increased sunlight intensity and solution temperature enhances the expression level of relative enzymes, which promotes the absorption of ${\text{HCO}}_{3}^{-}$ (Giordano and Beardall, 2005). The continually generated OH^−^ (Guan *et al*., 2017) reacts with ${\text{HCO}}_{3}^{-}$ to produce ${\text{CO}}_{3}^{2-}$ (Cheng *et al*., 2018a, b). Thus, the WRB gradually increases. Understanding the relationship between the WRB and environmental conditions is beneficial for facilitating actual production.

### Optimizing the Na_2_CO_3_/NaHCO_3_ weight ratio to improve pigment concentrations and trichome size of *Arthrospira* cells

**Figure 2:**
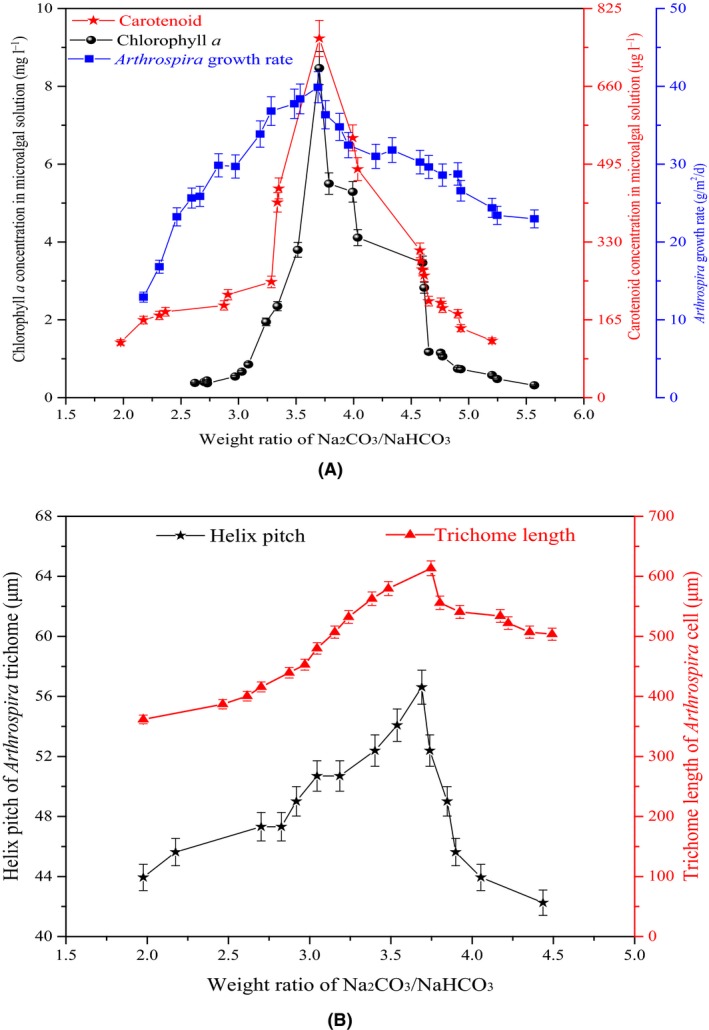
Effects of weight ratio of Na_2_ CO_3_/NaHCO_3_ on pigments and trichome of *Arthrospira* cells in residual solution after *Arthrospira platensis* growth in 660 m^2^ open raceway ponds. A. Effects on chlorophyll *a* and carotenoid concentrations. B. Effects on helix pitch and trichome length.

An increase in the WRB from 2.0 to 3.7 resulted in chl‐*a* and car‐d to increasing from 0.38 to 8.47 mg l^−1^ and 117 to 762 μg l^−1^, respectively (Fig. 2A). A further increase in the WRB to 5.6 caused chl‐*a* and car‐d to decrease from 8.47 to 0.32 mg l^−1^ and from 762 to 121 μg l^−1^, respectively. In addition, the *A. platensis* growth rate first increased to 39.9 g m^−2^ day^−1^ and then gradually decreased. There are four stages of *A. platensis* growth: adaptation, fast‐growth, stable‐growth and then the decline period. With the increased WRB, the Na_2_CO_3_ concentration and solution pH gradually increased, while the NaHCO_3_ concentration gradually decreased. When the WRB was 3.7, the solution pH was approximately 10.2 and the Na_2_CO_3_ and NaHCO_3_ concentrations were approximately 10.8 and 2.9 g l^−1^, respectively. Therefore, *A. platensis* was in a fast‐growth period. This explains the higher *A. platensis* growth rate. Chl‐*a*, the main pigment for photosynthesis, promotes the electron and ATP transfer rate (Fleming, 1967; Jansson, 1994; Wen *et al*., 2005; Babu and Ranganathan, 2014). As stated above, the solution pH increased with a higher WRB. NaHCO_3_ was continually consumed by the *A. platensis* cells, so the Calvin cycle was accelerated, thereby promoting the light reaction that supplies ATP (Yang *et al*., 2017). Therefore, more chl‐*a* was produced to support more light absorption. However, with the continued increase in the WRB, the solution pH was not suitable for microalgal growth, resulting in a decreased chl‐*a* concentration. Car‐d is the main source of vitamin A (Bassi *et al*., 2010). Increased car‐d would improve the light utilization ability of microalgal cells; therefore, with the increased WRB, car‐d first increased to improve photosynthesis and then decreased, corresponding to *A. platensis* growth.

The influence of WRB on the helix pitch and trichome length was evaluated (Fig. 2B). With the WRB increasing from 2.0 to 3.7, the helix pitch and trichome length increased from 44 to 57 μm and 362 to 613 μm, respectively. With a further WRB increase to 4.5, the helix pitch and trichome length decreased from 57 to 42 μm and from 613 to 503 μm, respectively. It is evident that with the growth of *A. platensis*, the helix pitch and trichome length increased (Toyoshima *et al*., 2015). The *A. platensis* cells, which are helical shaped, proliferates along the longitudinal axis. The increased WRB showed that when more ${\text{HCO}}_{3}^{-}$ was consumed, subsequently photosynthesis was increased. To satisfy the ATP requirements, microalgal cells elongate the helix pitch to increase the light contact area and light utilization efficiency (Ma and Gao, 2013). The improved photosynthesis promotes glucose accumulation and cell division, thereby increasing trichome length. However, with a further increase in WRB, the solution pH exceeded the cell's optimal range and the activity of the relative enzymes was reduced. Therefore, as photosynthesis decreased, the pigment concentrations, such as chl‐*a* and car‐d, decreased. Optimizing the WRB to improve the pigment concentrations of the microalgal solution and the trichome size of the *A. platensis* cells is beneficial to their growth. Furthermore, it is practical and feasible to evaluate the pigment concentrations and trichome size of *A. platensis* cells using the WRB in industry.

### Improving *Arthrospira* growth rate with a CO_2_ bicarbonation absorber process

**Figure 3:**
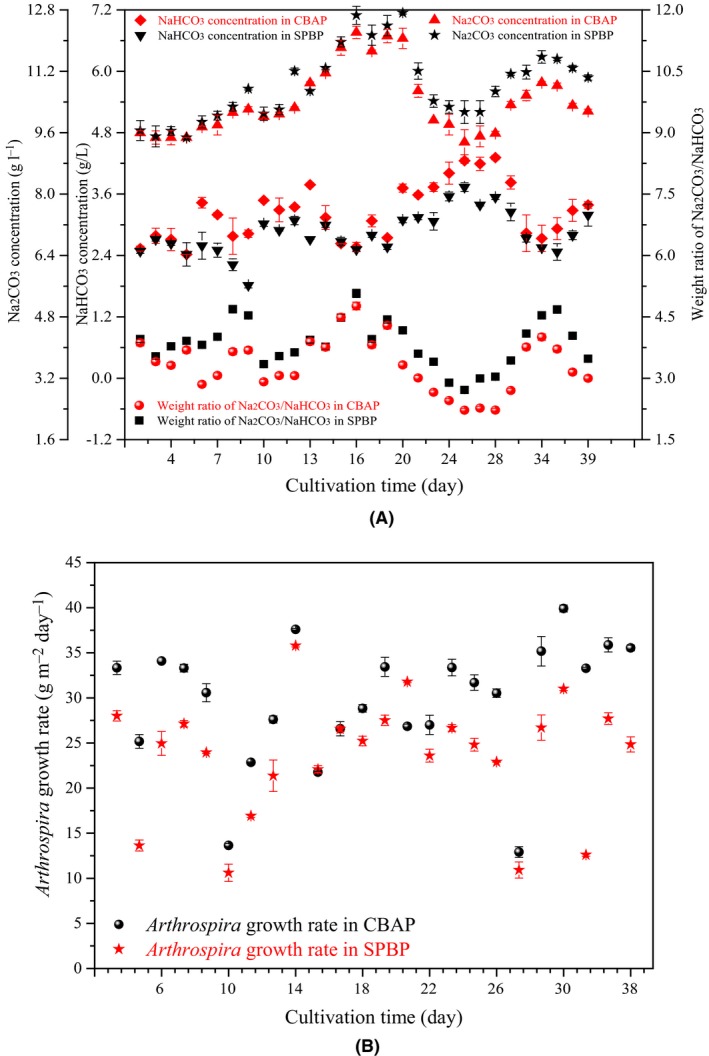
Comparison of dynamic *Arthrospira* biomass growth rate and Na_2_ CO_3_ **/**NaHCO_3_ weight ratio in residual solution between novel process with a CO_2_ bicarbonation absorber (CBAP) and traditional process with NaHCO_3_ plant by‐product (SPBP) in 660 m^2^ open raceway ponds. A. Comparison of Na_2_ CO_3_/NaHCO_3_ weight ratio in residual solution. B. Comparison of dynamic *Arthrospira* biomass growth rate.

In the CBAP, the average NaHCO_3_ concentration increased by 14.6% while the Na_2_CO_3_ concentration decreased by 4.0% (Fig. 3A). As a result, the average WRB of the CBAP (3.34) was 13.3% lower than in the SPBP (3.85). This may be because the NaHCO_3_ plant by‐product is in a solid state and needs time to dissolve in the raceway pond. Normally, the NaHCO_3_ by‐product is added to the raceway pond in a stationary place, so the NaHCO_3_ in the raceway pond is not well mixed. However, the NaHCO_3_ solution in the CBAP raceway pond was in a liquid state and was transferred by a porous pipe, which helps the mixing of NaHCO_3_. Since almost all of the CO_2_ gas was reacted with NaHCO_3_, the Na_2_CO_3_ concentration was very low in the CBAP. Therefore, the NaHCO_3_ concentration in the CBAP was higher than the Na_2_CO_3_ concentration in the SPBP. In addition, the microalgal NaHCO_3_ and Na_2_CO_3_ concentrations fluctuated day by day as a result of the varying daily temperatures and sunlight intensity. Therefore, the microalgal absorption ability of NaHCO_3_, as well as the CO_2_ transfer efficiency and NaHCO_3_ by‐product dissolution level, was different everyday, especially for this large‐scale application.

The average *A. platensis* growth rate in the CBAP (29.6 g m^−2^ day^−1^) was 25.3% higher than that in the SPBP (23.6 g m^−2^ day^−1^) (Fig. 3B). The above results fully demonstrate that the microalgal NaHCO_3_ and Na_2_CO_3_ concentrations co‐affect the *A. platensis* growth rate. Optimizing the WRB is helpful for improving the *A. platensis* growth rate. This may be because the higher NaHCO_3_ concentration contributes to *A. platensis* growth. The special CCM in the microalgal cells maintains a high growth rate even under limited CO_2_ concentrations. As a result of the CCM, ${\text{HCO}}_{3}^{-}$ is the dominant ion type used for the Calvin cycle, so a higher NaHCO_3_ concentration is beneficial for the *A. platensis* growth rate. However, the NaHCO_3_ concentration should be maintained within a certain range because of the imbalance of the light and dark reaction rates. Over‐assimilated NaHCO_3_ consumed more ATP, thus reducing the reaction rate of the Calvin cycle. NaHCO_3_ and Na_2_CO_3_ act as a ‘buffer pair’ in the *A. platensis* cell, which facilitates transportation of the NaHCO_3_ by the carbonic anhydrase enzyme. Moreover, the WRB is closely connected with the solution pH, which dominates the enzymatic activity of the microalgal cells. When the WRB is approximately 3.7, the enzymatic activity involved in the ion active transportation (e.g. carbonic anhydrase and RubisCO (Amc *et al*., 2017)) could be maintained at a high level. Therefore, the electron transport rate and light photon transfer efficiency of the Photosystem II are accelerated and the Calvin cycle is strengthened. As a result, the *A. platensis* growth rate is improved. Finally, reducing the WRB with a CO_2_ bicarbonation absorber to improve the *A. platensis* growth rate is an acceptable technique.

## Experimental procedures

### A novel industrial *Arthrospira platensis* cultivation process with a CO_2_ bicarbonation absorber

**Figure 4:**
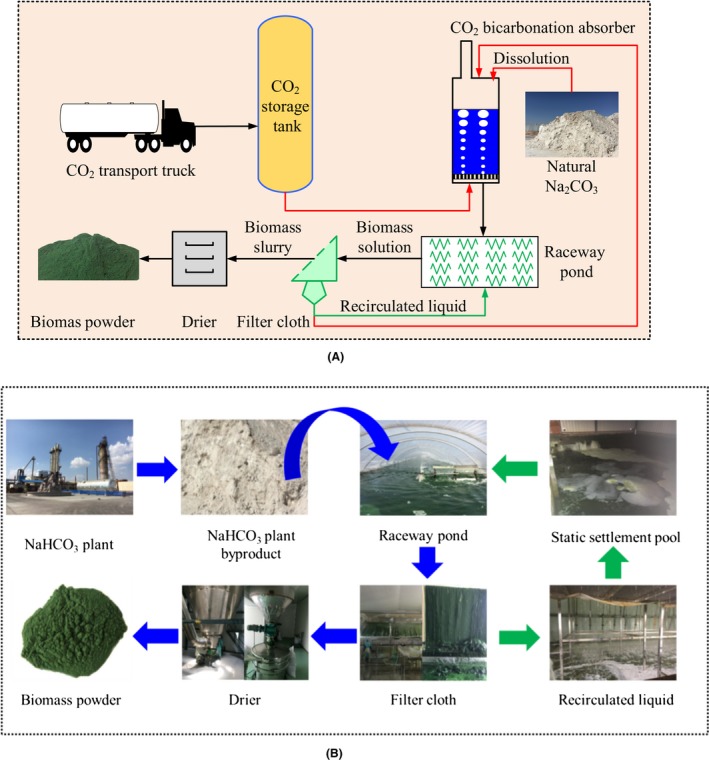
Comparison in industrial *Arthrospira platensis* cultivation between novel process with a CO_2_ bicarbonation absorber (CBAP) and traditional process with NaHCO_3_ plant by‐product (SPBP). A. Novel process in industrial *Arthrospira* cultivation with a CO_2_ bicarbonation absorber. B. Traditional process in industrial *Arthrospira* cultivation with NaHCO_3_ plant by‐product.

The novel industrial *A. platensis* cultivation process using a CO_2_ bicarbonation absorber is outlined in Fig. 4A. Briefly, purified CO_2_ gas was delivered by a CO_2_ transport truck and then stored in a CO_2_ storage tank, which was constructed along the raceway pond. The reaction between the CO_2_ gas and Na_2_CO_3_ solution was conducted in a sealed CO_2_ bicarbonation absorber (CBA). The reaction pressure of the CBA was 0.3 MPa, and the volume ratio of the Na_2_CO_3_ solution in the CBA was 60% (Guo *et al*., 2019). The Na_2_CO_3_ solution was a mixture of recirculated liquid (8–12 g l^−1^Na_2_CO_3_) and natural soda (20–30% Na_2_CO_3_). The dimensions of the CBA were φ1.6 × 3 m. Natural soda (50 kg) was dissolved daily into the recirculated liquid, which was injected into the CBA. CO_2_ gas was continually aerated into the CBA. The entire Na_2_CO_3_ solution was reacted with NaHCO_3_ for 90–120 min. The reacted NaHCO_3_ solution flowed to the raceway pond to satisfy the growth requirements of the *A. platensis* cells. The dimensions of the raceway pond were 110 × 6 m. The solution depth was 31.2 cm. After 4 days of cultivation, the *A. platensis* solution was harvested using a filter cloth. Following filtration, most of the recirculated liquid flowed back to the raceway pond, while some was injected into the CBA. The *A. platensis* slurry was dried with a dryer at 220°C and −0.2 MPa. After 60 min, the produced biomass powder had a moisture content < 10% and an ash content < 7%. The culture medium was composed of 5.0 g m^−2^ day^−1^ NaNO_3_, 0.45 g m^−2^ day^−1^ MgSO_4_, 0.3 g m^−2^ day^−1^ FeSO_4_, 0.15 g m^−2^ day^−1^ Na_2_EDTA, 2.6 g m^−2^ day^−1^ KCl, 0.55 ml m^−2^ day^−1^ H_3_PO_4_ and 15 g m^−2^ day^−1^ NH_4_HCO_3_. The experiments were conducted from the month of June to July with a light‐night cycle of 12:12. The whole system is defined as the CBA process (CBAP).

### The traditional industrial *Arthrospira platensis* cultivation process with NaHCO_3_ plant by‐product

The traditional industrial *A. platensis* cultivation method uses NaHCO_3_ plant by‐products as the carbon source (Fig. 4B). During actual cultivation, approximately 200 kg of NaHCO_3_ by‐product was added to the raceway pond at the inoculation time. On the 4th day of cultivation, the *A. platensis* biomass was harvested with a filter cloth (pore diameter −75 μm). *A. platensis* cells with a larger trichome length were collected as a slurry and then placed in the dryer, while the small *A. platensis* cells were recirculated back into the static settlement pool. Freshwater was added to the static settlement pool to compensate for water evaporation and residual solution in the pipeline. The biomass powder was produced using the dryer as described above. The recirculated liquid in the static settlement pool could be reused and pumped to the raceway pond. This system is defined as the SPBP.

In this study, microalgae were cultivated in four parallel raceway ponds. Two were operated under the CBAP, and the other two were operated under the SPBP. The cultivation cycle for both processes was 4 days. The first 3 days were for cultivation and the 4th day was for harvest and inoculation. We conducted 10 cultivation cycles to eliminate experimental error.

### Analytical methods

The concentrations of HCO_3_^−^ and CO_3_^2−^ in the *A. platensis* solution were measured using a double‐tracer technique (Couvert *et al*., 2017). The helix pitch and trichome length of *A. platensis* cells were measured with a microscope (XSP‐1C, China). Details of these methods have been described in a previous work (Cheng *et al*., 2018a).

Chlorophyll *a* (chl‐*a*) and carotenoid (car‐d) concentrations from *A. platensis* cells were also measured. During the experiment, 1 ml of the microalgal solution was filtrated using a vacuum pump. The filter paper with the collected microalgal cells was cut into small pieces and then placed in a centrifuge tube. Next, 5 ml of 100% (w/w) methyl alcohol was added to the centrifuge tube, mixed evenly and then placed in the dark for 30 min. Afterwards, the dissolved filter paper and microalgal residue was filtered. The subsequent filtrate was used to measure the absorbance at wavelengths of 480, 510, 652 and 665 nm. Chl‐*a* and car‐d concentrations (mg l^−1^) of the microalgal cells were determined using following formulas (Bednarczyk *et al*., 2015):

(1) $$\text{Chl-}a=16.29\times {\text{OD}}_{665\;\mathrm{nm}}-8.54\times {\text{OD}}_{652\;\mathrm{nm}}$$

(2) $$\text{Car-d}=7.6\times {\text{OD}}_{480\;\mathrm{nm}}-1.49\times {\text{OD}}_{510\;\mathrm{nm}}$$

The daily average sunlight intensity on the surface of the microalgal solution and microalgal solution temperature were measured using an illuminometer (TES Digital Lux Meter 1332A, China) and a thermometer. These measurements were made every 3 h at 7:00, 10:00, 13:00, 16:00 and 19:00.

A total of 1 l microalgal sample was taken at 7:00 and 19:00 daily, washed thrice and dried afterwards at 90°C for 24 h. The dry weight of the sample was measured to obtain the microalgal density *w* (g l^−1^). All experiments were conducted twice. The *A. platensis* growth rate *x* was calculated as follows:

(3) $$x=\left(w_{19:00}-w_{7:00}\right)\times 0.312\;m\times 1000\;(g\;m^{-2}\;{\mathrm{day}}^{-1})$$

## Summary

In this study, experiments were conducted in four 660 m^2^ raceway ponds for approximately 1 month to improve the CO_2_ fixation rate of *A. platensis*. Two large‐scale cultivation methods for *A. platensis* were first and thoroughly introduced. The CBAP was introduced as an alternative method to improve *A. platensis* growth. In the CBAP, pure CO_2_ and natural soda were used to improve NaHCO_3_ concentrations. These optimized operational conditions were introduced in a previous work (Chen *et al*., 2016). Those results show that the average ${\text{HCO}}_{3}^{-}$ concentration increased by 14.6% while the WRB decreased by 13.3% in the CBAP raceway pond. Additionally, the CBAP system is more economical and sustainable than SPBP. The NaHCO_3_ by‐product costs approximately $40/ton with a consumption of 200 kg day^−1^. Natural soda and CO_2_ gas cost approximately $10/ton and $70/ton with consumption rates of 50 and 12 kg day^−1^, respectively, which saved 83% of the operation cost. Moreover, the CBAP system can provide sustainable ${\text{HCO}}_{3}^{-}$ for the microalgae growth, which would be affected by the NaHCO_3_ content and dissolution rate of NaHCO_3_ in the SPBP system. Therefore, the CBAP is considered a promising method for the large‐scale cultivation of *A. platensis*.

The contributions of the present work are threefold: (i) we determined that the higher microalgal growth rate during CBAP was due to the lower initial WRB; (ii) we found that increased sunlight intensity, solution temperature and pH all resulted in enhanced cell growth that corresponded to more ${\text{HCO}}_{3}^{-}$ absorption and ${\text{CO}}_{3}^{2-}$ release, thus slightly promoting the WRB in the residual solution; and (iii) we found that the biomass growth rate first increased and then decreased with increasing WRB, causing the cell pigments (chlorophyll *a* and carotenoid) and trichome size (helix pitch and trichome length) to first increase and then decrease. However, there are still many limitations of this work. For example, the key mechanism by which the WRB affects *A. platensis* growth is still not understood. It is necessary to investigate the cellular structure and the mechanism by which ${\text{HCO}}_{3}^{-}$ crosses the cell membrane.

## Conclusion

Conversion of NaHCO_3_ to Na_2_CO_3_ using *A. platensis* cells was investigated in 660 m^2^ raceway ponds. Increased sunlight intensity, solution temperature and pH resulted in promotion of the Na_2_CO_3_/NaHCO_3_ weight ratio in the residual solution. The biomass growth rate was peaked at the Na_2_CO_3_/NaHCO_3_ weight ratio of 3.7. Correspondingly, cell pigments and trichome size arrived at a maximum state. A 13.3% lower Na_2_CO_3_/NaHCO_3_ weight ratio resulted in an increased biomass growth rate of 25.3% when using a novel CO_2_ bicarbonation absorber, compared to that with a traditional NaHCO_3_ plant by‐product.

## Conflict of interest

None declared.
